# Skeletal muscle ribosome analysis: A comparison of common assay methods and utilization of a novel RiboAb antibody cocktail

**DOI:** 10.14814/phy2.70173

**Published:** 2025-01-07

**Authors:** Joshua S. Godwin, J. Max Michel, C. Brooks Mobley, Gustavo A. Nader, Michael D. Roberts

**Affiliations:** ^1^ School of Kinesiology Auburn University Auburn Alabama USA; ^2^ Department of Kinesiology and Huck Institutes of the Life Sciences The Pennsylvania State University University Park Pennsylvania USA

**Keywords:** electrophoresis, fluorometry, RiboAb, ribosome pelleting, total RNA, UV–Vis

## Abstract

While total RNA concentrations putatively represent ribosome content, there is a need to homologize various quantification approaches. Thus, total RNA concentrations ([RNA]) provided through UV–Vis spectroscopy (UV), fluorometry‐only (Fluor), and fluorometry‐based microfluidic chip electrophoresis (MFGE) were examined in C2C12 myotubes and mouse skeletal muscle to determine if values aligned with [18S + 28S rRNA] (i.e., criterion ribosome metric). A novel antibody cocktail (termed *RiboAb*) was also tested and compared to [18S + 28S rRNA] in these models. In myotubes, 24‐h IGF‐1 treatments increased [18S + 28S rRNA] (~2.0‐fold) and [RNA] based on UV (~1.9‐fold), Fluor (~2.3 fold), and MFGE (~2.1‐fold). In C57BL/6 mice, 10 days of mechanical overload (MOV) elevated plantaris [18S + 28S rRNA] (~1.7‐fold) and [RNA] according to UV (~1.5‐fold), Fluor (~1.6‐fold), and MFGE (~1.8‐fold). Myotube and mouse plantaris RiboAb levels were significantly higher with IGF‐1 treatments and MOV, respectively, versus controls (1.3‐fold and 1.7‐fold, respectively), and values correlated with [18S + 28S rRNA] (*r* = 0.637 and *r* = 0.853, respectively, *p* ≤ 0.005). UV, Fluor, and MFGE [RNA] are seemingly valid surrogates of cell/tissue ribosome content, although each method has advantages (e.g., ease of use) and disadvantages (e.g., magnitudes of bias) discussed herein. Finally, the RiboAb cocktail may also represent ribosome content, although this should be further explored in other models.

## 1 INTRODUCTION

Ribosomes are macromolecules that reside in all living cells and catalyze protein synthesis through amino acid peptide bond formation (Green & Noller, 1997). Since early reports showing that a growth stimulus increases ribosome biogenesis in myotubes (Nader et al., 2005), there has been a burgeoning research interest in determining how exercise, aging, and diseased states affect skeletal muscle ribosome content (Chaillou et al., 2014; Jiao et al., 2023; Mesquita et al., 2021).

Several laboratories have reported total RNA concentrations obtained through UV–Vis spectroscopy (absorption maximum of 260 nm) to represent tissue ribosome content (Adams et al., 2004; Godwin et al., 2024; Hammarstrom et al., 2022; Haun et al., 2019; Mobley et al., 2018; Nakada et al., 2016; von Walden et al., 2012, 2016) given that the total RNA pool is comprised of 80%–85% ribosomal RNA (Hirsch, 1967). However, other methods for tissue RNA quantification exist including the use of fluorometric dyes alone (Makhnovskii et al., 2020; O'Reilly et al., 2021) or RNA dyes in tandem with microfluidic chip electrophoresis (MFGE) to delineate total RNA concentrations and/or the relative concentrations of the 28S and 18S rRNAs (Figueiredo et al., 2015; Stec et al., 2016). While less commonly employed due to the hardware and technical expertise needed, sucrose gradient density‐based ultracentrifugation techniques can be used to pellet cellular or tissue ribosomes (Lee & Kim, 2022). These methods can be combined with downstream UV–Vis spectroscopy or fluorometric methods to more accurately determine alterations in skeletal muscle ribosome content. Moreover, western blotting can be used to interrogate ~80 mammalian ribosomal proteins, albeit no literature to date has established if the relative content of one or multiple proteins correlates with changes in tissue ribosome content.

Muscle cell growth/hypertrophy can be stimulated in vitro with growth factors and in vivo with mechanical overload (e.g., resistance training in humans or synergist ablation in rodents), and it is well established that these growth stimuli increase ribosome content through the intricate process of ribosome biogenesis (Figueiredo & McCarthy, 2019; Kim et al., 2019; Roberts et al., 2023). However, although this phenomenon has been demonstrated in several rodent and human studies (Adams et al., 2004; Godwin et al., 2024; Hammarstrom et al., 2022; Haun et al., 2019; Mobley et al., 2018; Nakada et al., 2016; von Walden et al., 2012, 2016), a thorough comparison of methods commonly used for muscle cell/tissue ribosome quantification has not been performed. Therefore, we sought to determine how anabolic stimuli in C2C12 myotubes and murine plantaris muscle tissue affect cellular and tissue RNA concentrations, respectively, as assessed using UV–Vis spectroscopy (UV), fluorometry (Fluor), and fluorometry‐based microfluidic chip electrophoresis (MFGE). Note that we viewed 18S + 28S rRNA concentrations yielded from MFGE as our criterion metric for myotube and muscle tissue ribosome content as determined by pilot sucrose density pelleting experiments described herein. Additionally, we tested a novel ribosomal protein antibody cocktail (termed *RiboAb*) to determine if resultant western blotting data aligned with total RNA quantification methods across the myotube and mouse experiments.

## 2 MATERIALS AND METHODS

### 2.1 Summary of experimental methods

**FIGURE 1:**
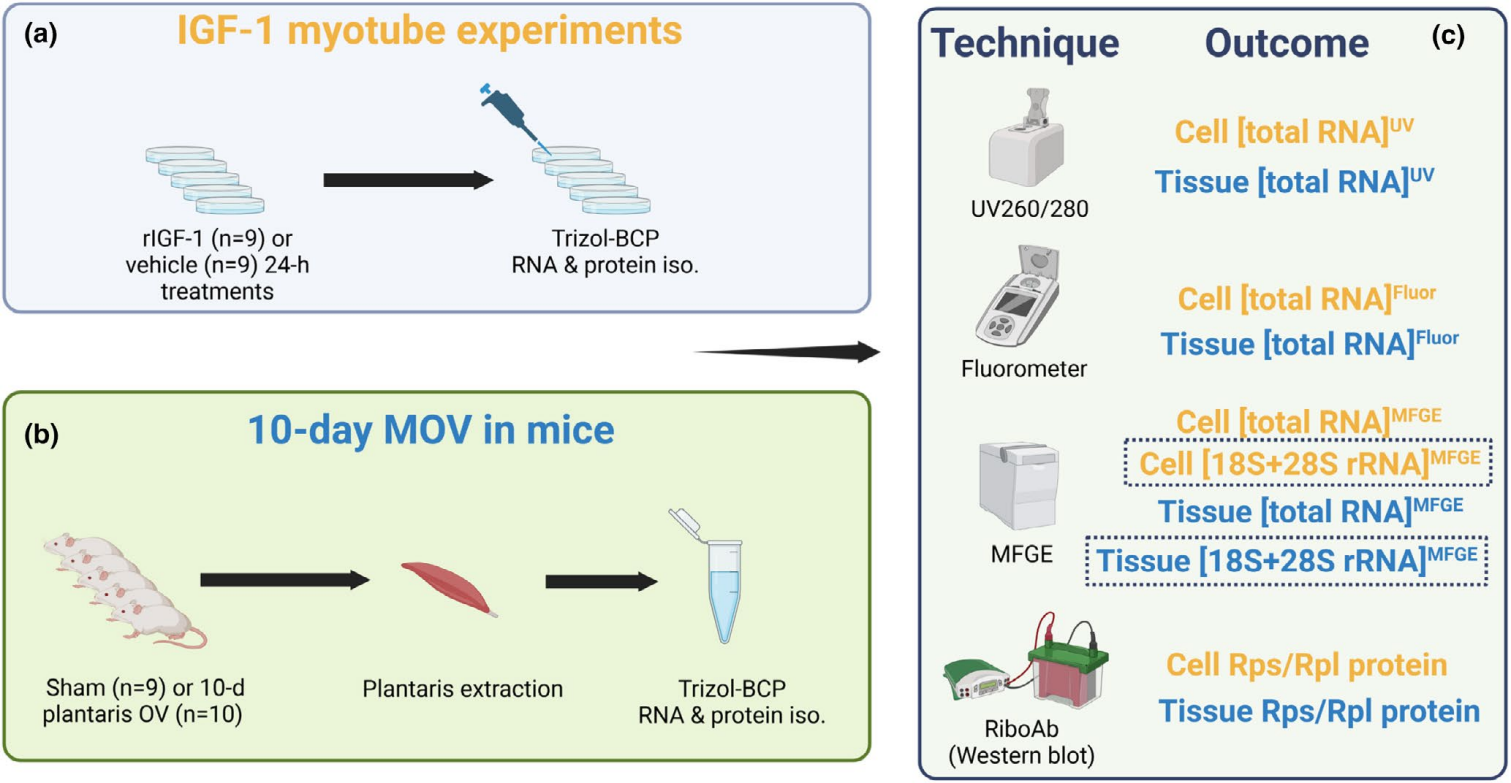
Summary of experimental methods. Whole cell RNA and protein isolates were obtained from C2C12 myotubes treated for 24 h with or without mouse recombinant insulin‐like growth factor‐1 (IGF‐1, 200 ng/mL) (a). Plantaris RNA and protein isolates were also from mice undergoing Sham (*n* = 9) or 10 days of mechanical overload (MOV, *n* = 10) via synergist ablation (b). RNA quantification methods for in vitro and MOV experiments are presented in panel (c). Note, we considered cell and tissue microfluidic RNA gel electrophoresis [28S + 18S rRNA] as the criterion metric for ribosome content (denoted with dashed boxes throughout) given the results from our ribosome pelleting experiments (summarized in Figure 2). BCP, 2‐bromochloropentane; Flour, fluorometric; MFGE, microfluidic RNA gel electrophoresis; Rpl, large subunit ribosomal protein; Rps, small subunit ribosome protein; UV, ultraviolet. Schematic was drawn using Biorender.com.

Figure 1 contains a summary of experimental methods aside from the initial C2C12 myotube ribosome pelleting experiments (described in the next section). Our first aim was to examine if an anabolic stimulus (IGF‐1) increased C2C12 ribosome content through obtaining 18S + 28S rRNA concentrations and total RNA concentrations using the UV, Fluor, and MFGE techniques. We next examined if an anabolic stimulus in vivo (MOV via synergist ablation) increased plantaris 18S + 28S rRNA concentrations and total RNA concentrations using the same three techniques. In both experiments, we also examined if RiboAb data aligned with 18S + 28S rRNA concentrations (i.e., our criterion metric of ribosome content as discussed in the next paragraph).

### 2.2 C2C12 myotube experiments for ribosome pelleting

Ribosome pelleting from C2C12 myotube lysates was performed to define a criterion metric for cell/tissue ribosome content as well as to determine if the RiboAb cocktail formulated to detect multiple ribosomal proteins reflected changes in cell/tissue ribosome content. For these experiments, myotubes were generated as described in the following section with the only difference that myotubes were grown on 100 mm plates to generate more cell matter for ultracentrifugation. Cell harvesting involved a PBS wash followed by 1 mL of a homemade lysis buffer containing a *base buffer* (50 mM Tris–HCl in DEPC‐treated water, pH 7.4; 250 mM KCl; 25 mM MgCl_2_; all ingredients from VWR, Radnor, PA, USA) and the following chemicals: (i) 0.25 mM dithiothreitol (VWR), (ii) 1 mg/mL cycloheximide (Sigma, Saint Louis, MO, USA; cat #: 01810), 800 U RNaseIN (Promega, Madison, WI, USA; cat #: N2111), and 0.5% Triton‐X 100 (VWR). Myotubes were scraped using rubber policemen to collect lysates into 1.7 mL microtubes. Following lysis on ice via tight‐fitting microtube pestles, samples were centrifuged at 4°C for 10 min to remove insoluble debris. Resultant supernatants were collected and placed atop either 20%, 30%, or 40% sucrose solutions (w:v in the base buffer described above) contained within 13.2 mL polyallomer tubes (Seton Scientific, Petaluma, CA, USA; cat #: 5030). Polyallomer tubes were then weighed on an analytical scale to ensure balance during ultracentrifugation, placed in a 2°C precooled swinging bucket ultracentrifuge rotor (Thermo Fisher Scientific, catalog #: TH‐641), and centrifuged at 100,000 ***g*** (2°C) for 1 or 3 h. Following ultracentrifugation, polyallomer tubes were placed in an ice bucket, and the top 1 mL (termed *top fraction*) was carefully removed with a pipettor and placed in a 2 mL microtube containing 1 mL Trizol (VWR). The remaining sucrose was carefully poured/discarded, 1 mL Trizol was pipetted onto ribosome pellets, and resultant slurries were transferred to fresh 1.7 mL microtubes. RNA and protein were isolated from the top fraction as well as RNA pellets using the Trizol‐bromochloropropane (BCP, Sigma; cat #: 241660) methods described by Wen et al. (2020). Additionally, RNA from both fractions was resuspended in 30 μL of DEPC‐treated water, and protein from both fractions was prepared for western blotting following the DC protein assay described later in the methods.

**FIGURE 2:**
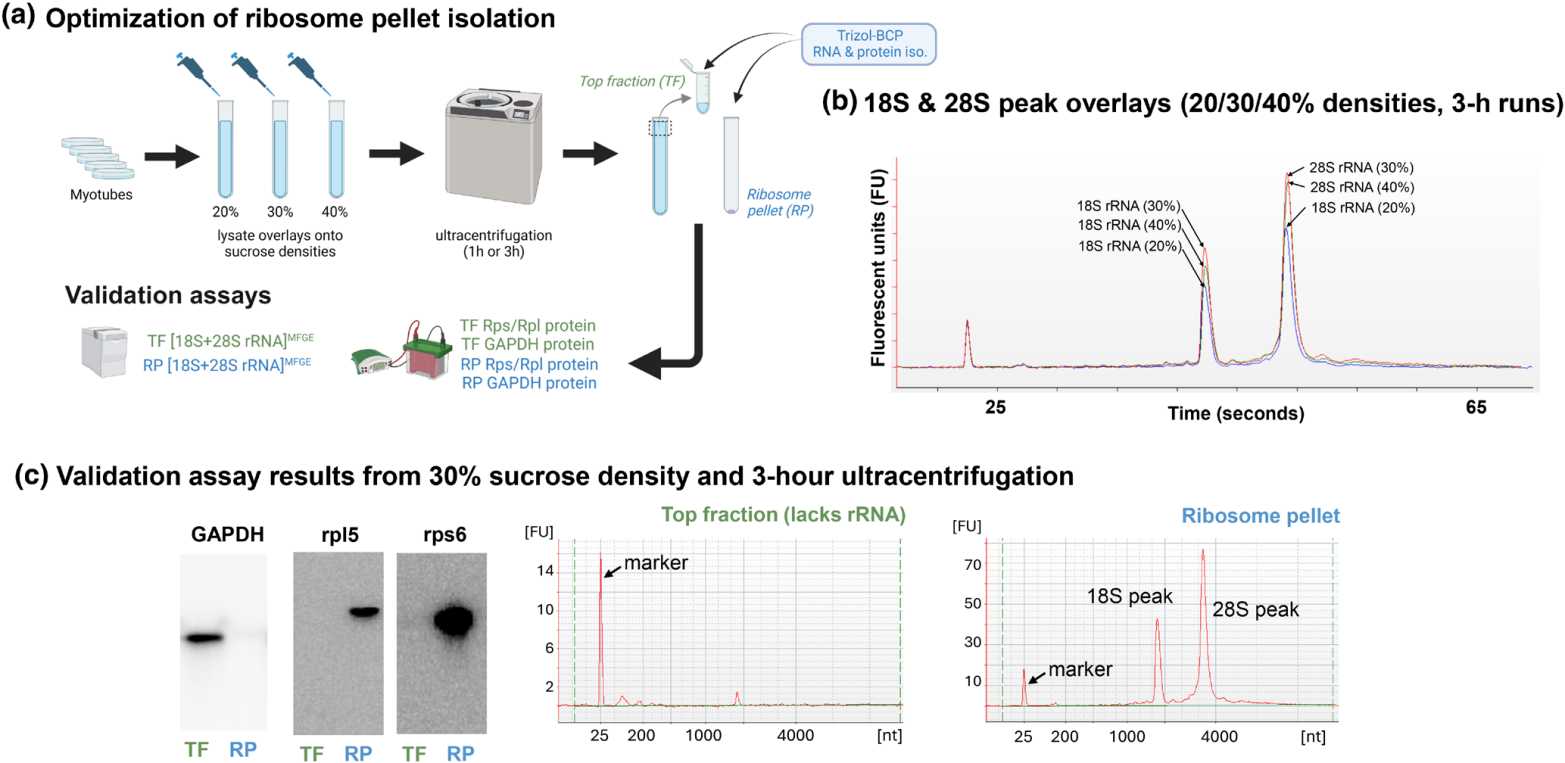
Ribosome pelleting using C2C12 myotube lysates. Panel (a) (drawn using Biorender.com) demonstrates the ribosome pelleting experimental workflow to obtain top fraction (non‐ribosomal proteins) and ribosome pellets from C2C12 myotubes. Panel (b) shows that 30% sucrose gradient (100,000 g spin time at 3 h) yielded highest 18S and 28S rRNA peaks compared to 20% and 40% gradients. Panel (c) shows that the 30% sucrose gradient (100,000 g spin time at 3 h) yielded a pure ribosome pellet evidenced by the presence of GAPDH protein only in the top fraction (TF) as well as the presence of rpl5/rps6 proteins and 18S&28S rRNAs only in the ribosome pellet fraction (RP). Given that the 18S&28S rRNAs were exclusively localized to the ribosome pellet, we considered 18S + 28S rRNA concentrations as our criterion metric for ribosome content throughout.

The experimentation of different sucrose concentrations and ultracentrifugation run times ensured that ribosome pelleting was successful. Key experimental outcomes we were aiming for included: (i) 28S and 18S ribosomal RNAs being largely absent from the top fraction and enriched in the ribosome pellet, (ii) the presence of a non‐ribosomal protein (GAPDH) being enriched in the top fraction and largely absent from the ribosome pellet, and (iii) ribosomal proteins being enriched in the ribosome pellet and largely absent from the top fraction. RNA from the top fractions and ribosome pellets were analyzed using fluorometric‐based microfluidic chip electrophoresis. 1‐h run times did not pellet ribosomes (*data not shown*), whereas 3‐h ultracentrifuge runs did as determined by the presence of 18S and 28S rRNAs in the pellet fraction (Figure 2b). Notably, the 30% sucrose density condition yielded the highest 18S and 28S peaks. Given Figure 2b results, we immunoblotted for GAPDH as well as ribosomal proteins as described later in the methods. Figure 2c indicated that 30% sucrose gradients with a 3‐h ultracentrifugation run time yielded top fractions with GAPDH protein and without 18S/28S rRNAs or ribosomal proteins. These results gave us confidence that 18S + 28S rRNA concentration was likely suitable as our criterion ribosome content metric.

### 2.3 C2C12 myotube IGF‐1 experiments

Low passage (passage 3–5) immortalized C2C12 myoblasts (ATCC; Manassas, VA, USA) were used for experiments in a humidified incubator set to 37°C using 5% CO_2_‐95% room air. Experimentation began with myoblasts being seeded onto six‐well plates (100,000 cells per mL) in growth medium (GM) containing Dulbecco's modified Eagle's Medium (DMEM; Corning, Corning, NY, USA) supplemented with 10% fetal bovine serum (Avantor® Seradigm, VWR; cat #: 89510–182), 1% penicillin/streptomycin (VWR; cat #: 97062–806), and 0.1% gentamycin (VWR; cat #: 97061–372). Once cells reached confluency (~85%–90%), differentiation was induced by switching to differentiation medium (DM), which consisted of DMEM supplemented with 2% horse serum (Corning; cat #: 35‐030‐CV), 1% penicillin/streptomycin, and 0.1% gentamycin. Once myotube formation was deemed visually sufficient (~7 days), cells were treated for 24 h with either phosphate buffered saline (PBS; CTL, *n* = 9 replicates) or 200 ng/mL of recombinant mouse IGF‐1 resuspended in PBS (IGF‐1, *n* = 9 replicates; R&D Systems, Minneapolis, MN, USA; cat #: 791‐MG‐050). Following treatments, cells were washed once with PBS, and 500 μL of Trizol (Millipore Sigma, Burlington, MA, USA; cat #: 93289) was added to wells. Plate wells were then scraped using rubber policemen to collect lysates into microtubes, and samples were stored at −80°C until protein and RNA isolation.

### 2.4 Synergist ablation mouse experiments

Mouse experiments were conducted in accordance with the institutional guidelines for the care and use of laboratory animals as approved by the Animal Care and Use Committee of the University of Kentucky (protocol #: 2008–0291). All mice were housed in a climate‐controlled room and maintained on a 14:10 h light–dark cycle, food (Teklad 2918 protein rodent diet; Envigo, Indianapolis, IN, USA) and water consumption were allowed ad libitum. Surgical removal of synergist muscles (part of the gastrocnemius and the entire soleus) to mechanically overload (MOV) the plantaris muscle was performed as previously described (McCarthy et al., 2011). Briefly, adult (>4‐month‐old) wild‐type C57BL/6 mice (*n* = 10, 6 males and 4 females) were anesthetized with 3% isoflurane (with 1.5 L of O_2_ per minute) and placed in sternal recumbence where a longitudinal incision was made on the dorsal aspect of the lower hindlimb, and the tendon of the gastrocnemius muscle was isolated and used as a guide to excise the soleus and part of the gastrocnemius. The incision was then sutured, and the animals were allowed to recover in their home cages. Sham surgeries (*n* = 9, 4 males and 5 females) involved similar incision and suture procedures without the excision of muscles as described above. Ten days following surgeries, mice were anesthetized, euthanized via cervical dislocation, and the plantaris muscle was excised. Immediately following removal, the plantaris muscles were weighed, flash‐frozen in liquid nitrogen, and stored at −80°C until RNA and protein isolation using the Trizol‐BCP methods described by Wen et al. (2020).

### 2.5 RNA isolation and concentration assessments

Aqueous phase RNA was isolated from cell‐Trizol slurries using the modified Trizol‐bromochloropropane (BCP) protocol described by Wen et al. (2020). Mouse tissue teased of connective tissue (~20 mg) was placed in 1.7 mL tubes, and tight‐fitting pestles were used to homogenize in 1 mL of Trizol, and aqueous phase RNA was also isolated using the modified Trizol‐bromochloropropane (BCP) protocol described by Wen et al. (2020).

Following RNA isolations, RNA pellets yielded from cells and plantaris samples were resuspended in 30 μL of RNase‐free water and frozen at −80°C until RNA assays. The day of experimentation, total RNA concentrations were determined using the following methods: (i) UV, UV–Vis spectroscopy at an absorbance of 260 nm by using a NanoDrop Lite (Thermo Fisher Scientific, Waltham, MA, USA), (ii) Fluor, fluorometrically via a commercially available assay (Thermo Fisher Scientific, cat #: Q10210) measured with a Qubit Analyzer (Thermo Fisher Scientific), (iii) MFGE, fluorometric‐based microfluidic chip electrophoresis via a commercially available assay (Agilent, Santa Clara, CA, USA, cat #: 5067–1511) measured with the Agilent 2100 Bioanalyzer system. UV involved blanking the device with 1 μL of RNAse‐free water twice and then reading 1 μL duplicates from each sample consecutively. Fluor involved preparing a large working solution stock for each sample by diluting the fluorometric reagent in RNA assay buffer (1:200). For standard curve generation, 190 μL of working solution was combined with 10 μL of each Qubit RNA HS Standard (0 ng/μL and 100 ng/μL) in separate Qubit assay tubes. Duplicate sample measurements were performed by mixing 199 μL of working solution with 1 μL of each RNA sample in duplicate tubes. All tubes were vortexed for 2–3 s and incubated at room temperature for 2 min. Standard tubes were then placed and read in the Qubit Fluorometer, and sample tubes were read sequentially thereafter. Fluor RNA concentration readings were determined via calibration with standard readings and corrected for dilutions by the device. MFGE first involved decontaminating the machine's electrodes using RNase Away and nuclease‐free water. The RNA Nano gel matrix was then filtered and mixed with the dye concentrate according to manufacturer's specifications. The chip was prepared by loading 9 μL of gel‐dye mix into the designated wells, followed by 5 μL of RNA marker and 1 μL of either RNA ladder or sample (duplicate) into their respective wells. The chip was vortexed at 2400 rpm for 1 min and analyzed within 5 min. To mitigate freeze–thaw artifact, all methods were performed the same day for cells and same day for tissues, and RNA samples were thawed and kept on ice throughout.

### 2.6 Protein concentration assessments

To obtain a cellular phenotype (total protein per well) as well as determine cellular RiboAb content, protein was isolated from the organic phase of the Trizol‐BCP mix as described by Wen et al. (2020). Protein concentrations were then determined with the DC protein assay kit (Bio‐Rad, Hercules, CA, USA, cat #: 5000121) and absorbance readings at 700 nm using a microplate spectrophotometer (Biotek Synergy H1 hybrid reader; Agilent). Protein was also isolated from the organic phase of the Trizol‐BCP mix from the male mice only for RiboAb content determination as described by Wen et al. (2020). Protein concentrations were then determined via the RC DC Protein assay kit (Bio‐Rad).

### 2.7 RiboAb validation and western blotting methods for in vitro and mouse MOV samples

**FIGURE 3:**
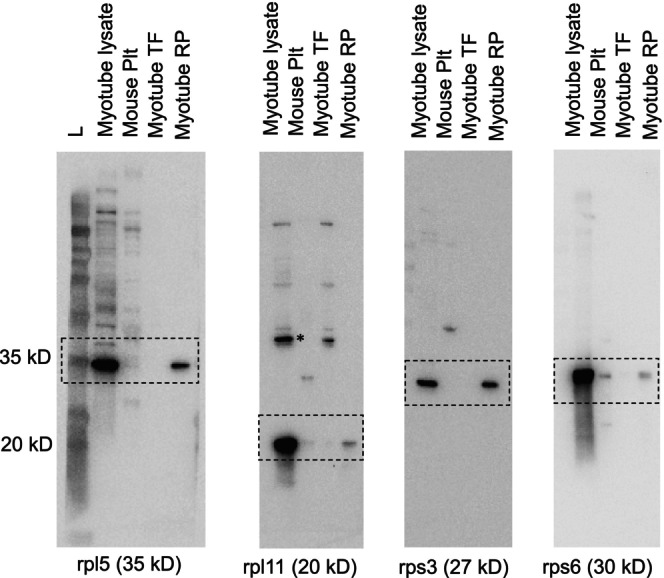
RiboAb validation. Immunoblotting results of rpl5, rpl11, rps3, and rps6 are presented for whole cell lysates, mouse plantaris lysates, top fraction (TF) of myotube isolates following ultracentrifugation, and ribosome pellet (RP) following ultracentrifugation. Symbols: Boxes represent target protein; asterisk for rpl11 indicate potential non‐specific band.

Upon confirmation that 30% sucrose with 3‐h ultracentrifugation run times yielded purified ribosome pellets from C2C12 myotubes (Figure 2), we developed our RiboAb cocktail (20 μL used at a dilution of 1:1000) containing 5 μL rpl5 (Cell Signaling, Danvers, MA, USA, cat #: 14568S), 5 μL rpl11 (Cell Signaling, cat #: 18163S), 5 μL rps3 (Cell Signaling, cat #: 9538S), and 5 μL rps6 (Cell Signaling, cat #: 2217). These four antibodies were chosen based on a few criteria. First, we aimed to develop a cocktail of readily and commercially available rabbit host antibodies for researchers aiming to adopt our RiboAb approach. Second, not only were rpl5 and rps6 exclusively present in the ribosome pellet of myotubes (seen in Figure 2), but additional western blotting experiments also indicated the lack of rpl11 and rps3 in the top fraction and enrichment of these proteins in the ribosome pellet (see Figure 3). Finally, a recent deep proteomics investigation on human skeletal muscle biopsies by our laboratory indicated that rps3/6 and rpl5/11 were moderately‐to‐highly abundant ribosomal proteins across participants (Roberts et al., 2024); hence, we posited that these targets are likely highly abundant in skeletal muscle across species.

For RiboAb CTL/IGF‐1 C2C12 myotube experiments, protein isolates from all 18 samples were standardized to 0.5 μg/μL following the RC DC assay. RiboAb MOV/Sham plantaris experiments were only performed for the 10 male mice due to lysate constraints with other ongoing laboratory projects, and protein isolates were standardized to 1.0 μg/μL following the DC assay. Western blotting preps were then loaded onto pre‐casted 4%–15% polyacrylamide gels (26‐well Criterion TGX gels; Bio‐Rad) and subjected to electrophoresis (180 V for 50 min). Proteins were then transferred to methanol‐preactivated PVDF membranes (Bio‐Rad) for 2 h at 200 mA. Gels were then Ponceau stained for 10 min, washed with diH_2_O for ~30 s, dried, and digitally imaged (ChemiDoc Touch, Bio‐Rad). Following Ponceau imaging, membranes were reactivated in methanol, blocked with non‐fat bovine milk for ~1 h, and washed 3 × 5 min in Tris‐buffered saline with Tween 20 (TBST). Membranes were then incubated with the RiboAb cocktail (1:1000 v/v dilution in TBST with 5% bovine serum albumin (BSA)) overnight. Following primary antibody incubations, membranes were washed 3 × 5 min in TBST and incubated for 1 h with HRP‐conjugated anti‐rabbit IgG (diluted 1:2000 v/v in TBST with 5% BSA; Cell Signaling, cat #: 7074). Membranes were finally washed 3 × 5 min in TBST, developed using chemiluminescent substrate (Millipore; cat #: ELLUF0100), and digitally imaged. RiboAb band densities were obtained using commercially available software (Bio‐Rad) and normalized to Ponceau densitometry values. Fold‐change values were then derived by dividing Ponceau‐normalized RiboAb band density values by the aggregate mean value of either the CTL myotubes (cell experiments) or Sham group (MOV experiment).

### 2.8 Statistics

Total RNA, 18S + 28S rRNA, and RiboAb outcomes were compared between CTL and IGF‐1 as well as MOV and Sham conditions using dependent and independent samples *t*‐tests, respectively. Bland–Altman plots were also performed for the in vitro and in vivo data, and associations throughout were performed using Pearson correlations. Data were plotted and analyzed in GraphPad Prism (v10.2.2), statistical significance was established as *p* ≤ 0.05, and all data throughout are expressed as mean and standard deviation values with individual data points.

## 3 RESULTS

### 3.1 An increase in ribosome content following IGF‐1 was consistently detected using different methodologies

**FIGURE 4:**
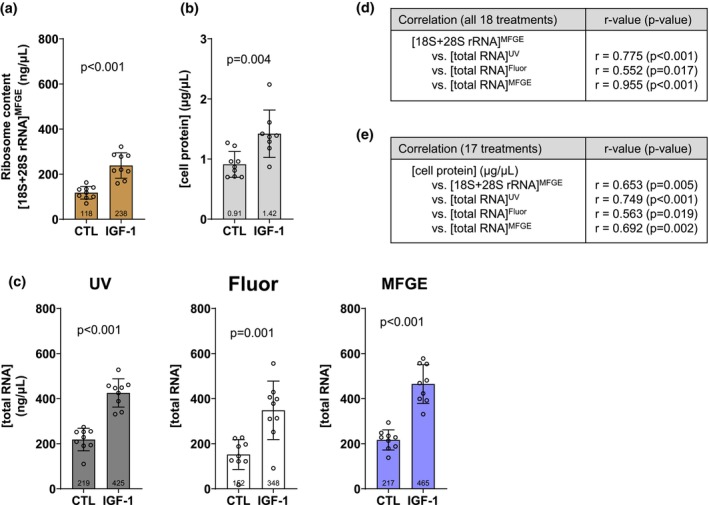
In vitro effects of IGF‐1 on myotube ribosome content assessed with various methods. Data for C2C12 myotubes treated without (CTL, *n* = 9 replicates) or with 200 ng/mL IGF‐1 (IGF‐1, *n* = 9 replicates) for 24 h. IGF‐1 increased myotube ribosome content assessed with our criterion outcome of 18S + 28S rRNA concentrations (a). IGF‐1‐induced anabolism as evidenced with increased protein content was also evident (b). Total RNA concentrations were also higher in IGF‐1‐treated myotubes as assessed by UV–Vis (UV), fluorometry (Fluor), and fluorometry in tandem with microfluidic chip electrophoresis (MFGE) (c). Data in panels (a–c) are presented as individual values overlayed on mean ± standard deviation bars. Panel (d) shows Pearson correlation data between the criterion ribosome content metric ([18S + 28S rRNA]) and total RNA assessed by each method. Panel (e) shows Pearson correlation data between the cellular phenotype and [18S + 28S rRNA] as well as total RNA assessed by each method.

Again, we viewed 18S + 28S rRNA concentrations as our criterion metric for myotube and muscle tissue ribosome content since myotube ribosome pelleting experiments indicated the 18S and 28S rRNAs were confined to the ribosome pellet (Figure 2). Myotubes treated with IGF‐1 presented significantly greater 18S + 28S rRNA concentrations compared to CTL (*p* < 0.001; Figure 4a), and this coincided with an anabolic phenotype of more cellular protein in IGF‐1‐treated versus CTL myotubes (*p* = 0.004; Figure 4b). IGF‐1‐treated myotubes also presented greater total RNA concentrations than CTL as assessed using UV (*p* < 0.001), Fluor (*p* = 0.001), and MFGE (*p* < 0.001) (Figure 4c). We next sought to correlate total RNA concentrations assessed with the three techniques to the criterion 18S + 28S rRNA concentration metric (Figure 4d) in all 18 individual treatments (*n* = 9 replicates per treatment group). Data yielded from all three techniques demonstrated significant positive correlations to 18S + 28S rRNA concentrations (*p* < 0.05). Finally, we correlated 18S + 28S rRNA concentrations as well as total RNA concentrations assessed with the three techniques to protein concentrations (i.e., cellular phenotype; Figure 4e). All four outcomes demonstrated significant positive correlations to protein concentrations (*p* < 0.05).

### 3.2 MOV promotes and increase in plantaris ribosome content as assessed using different methodologies

**FIGURE 5:**
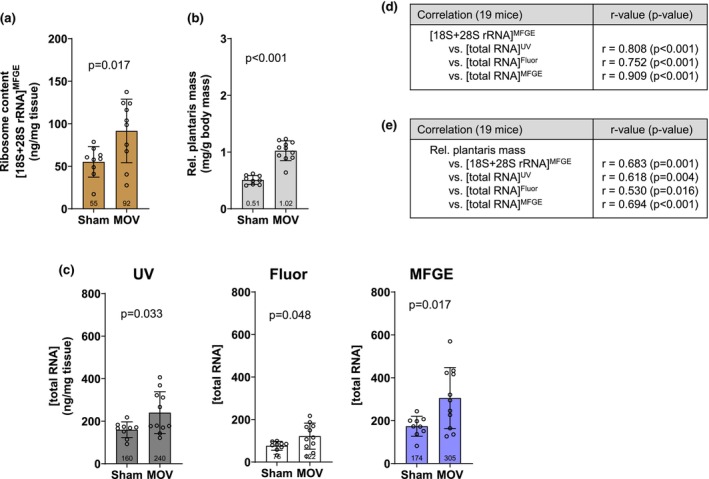
10‐day MOV effects on mouse plantaris ribosome content assessed with various methods. Data for mouse Sham plantaris (*n* = 9 mice) or following 10 days of mechanical overload (MOV, *n* = 10). MOV increased tissue ribosome content assessed with our criterion outcome of 18S + 28S rRNA concentrations (a). Tissue hypertrophy was also evident (b). Total RNA concentrations were also higher in MOV versus Sham as assessed by UV–Vis (UV), fluorometry (Fluor), and fluorometry in tandem with microfluidic chip electrophoresis (MFGE) (c). Data in panels (a–c) are presented as individual values overlayed on mean ± standard deviation bars. Panel (d) shows Pearson correlation data between the criterion ribosome content metric ([18S + 28S rRNA]) and total RNA assessed by each method. Panel (e) shows Pearson correlation data between the tissue phenotype (body mass‐normalized plantaris mass) and [18S + 28S rRNA] as well as total RNA assessed by each method.

MOV presented significantly greater 18S + 28S rRNA concentrations compared to Sham (*p* = 0.017; Figure 5a), and this coincided with a greater relative plantaris mass (*p* < 0.001; Figure 5b). Total RNA concentrations when assessed using UV (*p* = 0.033), Fluor (*p* = 0.048), and MFGE (*p* = 0.017) (Figure 5c) were also greater in MOV versus Sham. Again, we correlated total RNA concentration data yielded from the three techniques to the criterion metric of 18S + 28S rRNA concentrations (Figure 5d) in all 19 mice. Data from all three techniques demonstrated significant positive correlations to 18S + 28S concentrations (*p* < 0.05). 18S + 28S rRNA concentrations as well as total RNA concentrations assessed with the three techniques demonstrated significant positive correlations to relative plantaris masses (i.e., tissue phenotype; Figure 5e).

### 3.3 Duplicate performance characteristics of UV, Fluor, and MFGE

Given that duplicate total RNA concentration readings were performed for the in vitro and mouse MOV experiments, we calculated duplicate reliability characteristics for the UV, Fluor, and MFGE techniques (Table 1). Outcomes included coefficient of variation (CV), intraclass correlation (ICC_3,1_) and standard error of the measurement (SEM) as described by Weir (2005). For the in vitro experiment, data yielded from all three methods yielded exceptional reproducibility, albeit minor differences in reliability scores suggest that UV outperformed other methods. For the mouse experiment, data yielded from all three methods also yielded exceptional reproducibility; however, numerical differences suggest that Fluor and UV produced better reliability scores than MFGE.

**TABLE 1:** Reliability characteristics of total RNA concentrations determined in duplicate via UV, Fluor, and MFGE for C2C12 myotube IGF‐1 and mouse MOV experiments.

Outcome	CV (%)	ICC_3,1_	SEM ([])
Myotube data (17–18 samples)			
[total RNA]^UV^	5.2	0.979	24.9 ng/μL
[total RNA]^Fluor^	12.8	0.964	38.4 ng/μL
[total RNA]^MFGE^	6.9	0.935	52.6 ng/μL
Mouse MOV data (19 samples)			
[total RNA]^UV^	5.0	0.980	10.7 ng/μL
[total RNA]^Fluor^	4.3	0.992	3.9 ng/μL
[total RNA]^MFGE^	11.3	0.951	24.6 ng/μL

*Note*: [total RNA]^MFGE^ readings for one of the cell replicates yielded very low and nonsensical data suggestive of technical error. Thus, this value were removed from the analyses.

Abbreviations: CV, coefficient of variation; ICC_3,1_, intraclass correlation r‐values; SEM, standard error of the measurement.

### 3.4 Bland–Altman plots

**FIGURE 6:**
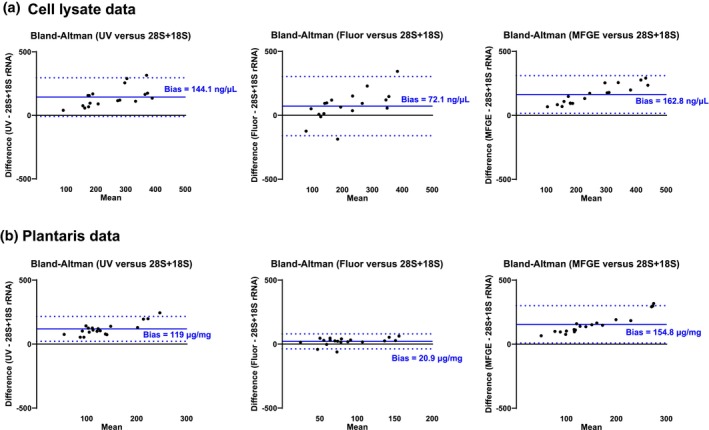
Bland–Altman plots of the UV, Fluor, and MFGE total RNA data. Plots for cell lysate total RNA data yielded by the three techniques in comparison to 28S + 18S rRNA yielded by MFGE (a). Plots for platnaris total RNA data yielded by the three techniques in comparison to 28S + 18S rRNA yielded by MFGE (b). Mean bias values are indicated in each plot by solid blue lines and upper and lower‐bound limits of agreement are presented as dashed lines.

Bland–Altman plots for in vitro data are presented in Figure 6a. Analyses revealed differing levels of agreement between the UV, Fluor, and MFGE methods compared to 28S + 18S rRNA (criterion measure). UV showed a mean bias of 144 ng/μL with 95% limits of agreement ranging from −7.9 to 296.1 ng/μL, Fluor demonstrated a smaller bias of 72.1 ng/μL with wider limits of agreement (−159.9 to +304.0 ng/μL), and MFGE demonstrated similar bias and limits of agreement to UV (162.8 ng/μL, −15.6 to +309.9 ng/μL). Although the smaller bias favors Fluor, the tighter limits of agreement observed with UV and MFGE suggest that these methods may provide more consistent measurements compared to Fluor when measuring cell lysate RNA concentrations.

Bland–Altman plots for in vivo data are presented in Figure 6b. While analyses also revealed differing levels of agreement between the three methods compared to 28S + 18S rRNA, results were not consistent with the in vitro data. Specifically, Fluor demonstrated the smallest bias (20.9 μg/mg) with narrower of limits of agreement (−37.6 to +79.5 μg/mg) compared to UV (119.0 μg/mg, 21.5–216.6 μg/mg) and MFGE (154.8 μg/mg, 9.1–300.4 μg/mg). Hence, unlike the in vitro data discussed in the prior paragraph, the smaller bias and limits of agreement with Fluor seemingly favor this method when measuring tissue RNA concentrations.

### 3.5 RiboAb validation

**FIGURE 7:**
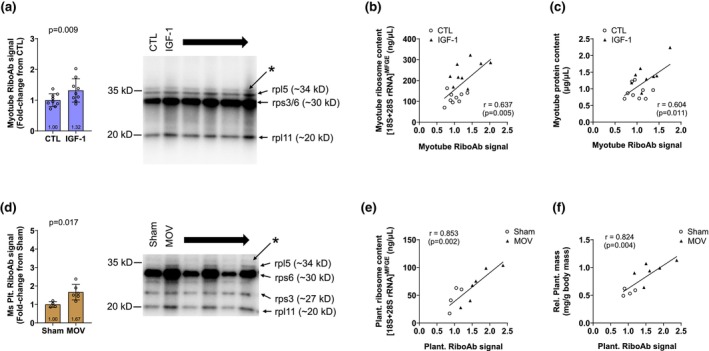
RiboAb outcomes with in vitro IGF‐1 treatments and MOV in a subset of mice. Densitometric RiboAb values were greater in IGF‐1‐treated (*n* = 9 replicates) versus CTL (*n* = 9 replicates) myotubes (a). Significant correlations with cellular ribosome content (i.e., [18S + 28S rRNA]) (b) as well as total protein content (i.e., cell phenotype) (c) were evident. Due to lysate constraints, only male mice were assayed for RiboAb. Notwithstanding, plantaris RiboAb values were greater in 10‐day MOV (*n* = 6 male mice) versus Sham mice (*n* = 4 male mice) (d). Significant correlations with tissue ribosome content (e) as well as relative plantaris masses (f) were evident. Data in panels (a, d) are presented as individual values overlayed on mean ± standard deviation bars. The rsp3/6 are likely overlaid in myotube lysates (extrapolated from Figure 6 results) and asterisks represent a band at ~37 kD which we believe is a non‐specific band yielded from the rpl11 antibody.

Figure 3 shows each individual RiboAb validated via Western blotting on myotube protein isolates, mouse plantaris protein isolates, protein isolated from the top fraction following ultracentrifugation (30% sucrose gradient, 3‐h run time), and protein isolated from the ribosome pellet (30% sucrose gradient, 3‐h run time). Results indicated that, aside from rpl11 showing a non‐specific band at 37 kD, all other antibodies yielded prominent bands at their putative molecular weights. Given that the myotube lysate was enriched with ribosomal proteins, signals from mouse plantaris protein isolates were relatively low. However, these signals were adequately expressed on separate membranes containing only mouse plantaris protein (see Figure 7). Finally, it is notable that each ribosomal protein was enriched in the ribosome pellet (but not top fraction) yielded by ultracentrifugation experiments.

### 3.6 RiboAb correlates with the increase in ribosome content during in vitro and in vivo hypertrophy

RiboAb levels were greater with 24‐h IGF‐1 treatments versus CTL (*p* = 0.009, Figure 7a), and there were significant correlations between the RiboAb levels and myotube 18S + 28S concentrations (*r* = 0.636, *p* = 0.005; Figure 7b) as well as cellular protein content (*r* = 0.604, *p* = 0.011; Figure 7c). MOV plantaris muscle (males only, *n* = 6) also exhibited a greater RiboAb levels versus Sham (males only, *n* = 4; *p* = 0.017, Figure 7d), and there were also significant correlations between the RiboAb signal and plantaris 18S + 28S concentrations (*r* = 0.853, *p* = 0.002; Figure 7e) as well as relative plantaris masses (*r* = 0.824, *p* = 0.004; Figure 7f). Lysate constraints precluded duplicate readings; thus, reliability statistics were not performed.

## 4 DISCUSSION

Multiple laboratories have used various techniques (namely the UV, Fluor, and MFGE methods) to determine how exercise, aging, and diseased states affect muscle ribosome content. However, a rigorous comparison between the three common techniques used to assess cellular/tissue total RNA concentrations is currently lacking. Stimulating myotube anabolism via IGF‐1 in vitro and 10 days of plantaris MOV in mice significantly increased total RNA concentrations as determined by the UV, Fluor, and MFGE techniques. Total RNA concentration data yielded by the three techniques across these experiments also exhibited significant associations with myotube/plantaris phenotypes as well as 18S + 28S rRNA concentrations, the latter being what we considered the criterion metric for cell and muscle tissue ribosome content. The implications of these and other findings are discussed in the following paragraphs.

Indeed, variations in RNA concentrations were observed between the techniques (notably, Fluor was consistently lowest), and this can likely be attributed to the fundamental differences in their measurement principles. UV measures nucleic acid wavelength absorption at 260 nm and can overestimate RNA concentrations due to its inability to discriminate between intact versus degraded RNA as well as free nucleotide, DNA, and protein contaminants. Although MFGE indicated low RNA degradation in virtually all cell and plantaris RNA (as evidenced by prominent 18S and 28S peaks), we cannot rule out that some degradation and/or slight DNA/protein contamination may have inflated UV readings. In contrast, Fluor and MFGE utilize RNA‐specific fluorescent dyes that selectively intercalate between the bases of RNA molecules and MFGE also employs microfluidic separation of nucleotides allowing for both the quantification and quality assessments of RNA. MFGE is the only technique that distinguishes between intact RNA and degradation products, potentially yielding lower concentration values compared to UV when degraded RNA is present. However, the consistently lower Fluor versus MFGE and UV values is difficult to reconcile and may be a limitation of the technique.

Interestingly, Bland–Altman (Figure 6) and reliability analyses (Table 1) indicate that UV provides the most consistent in vitro RNA concentration measurements whereas Fluor provides the most consistent in vivo RNA concentration measurements. However, aside from a relatively high bias yielded when comparing the UV [RNA] to [28S + 18S rRNA] data, we posit that estimating ribosome content via the UV method poses certain advantages. Most notably, performing UV duplicate readings per sample is very rapid (~10 min for 20 duplicates), and aside from needing a UV–Vis spectrophotometer, there is no need to purchase specialized reagents as with the Fluor of MFGE methods. Compared to Fluor readings, it is also notable that UV readings of both myotube and plantaris samples showed higher correlations with their corresponding 18S + 28S rRNA concentrations and phenotype outcomes. Hence, not only does the current data align with several prior studies positing that [total RNA]^UV^ represents the cell/tissue ribosome pool (Adams et al., 2004; Hammarstrom et al., 2022; Haun et al., 2019; Mobley et al., 2018; Nakada et al., 2016; von Walden et al., 2012, 2016), but the current findings also suggest that the UV–Vis technique is robust and highly reproducible.

Finally, the newly proposed RiboAb technique warrants further discussion. The motivation for developing RiboAb was due to the challenging nature of quantifying cellular or tissue macromolecules in general. The 20S proteasome, another macromolecule of interest to muscle biologists, contains ~28 subunits (Tanaka, 2009), and there are cocktails containing antibodies against several of these subunits available for researchers to estimate cellular/tissue proteasome content (Michel et al., 2023; Moberg et al., 2017). Our sucrose ultracentrifugation experiments indicated that the four rps/rpl proteins used for the RiboAb cocktail predominantly localize to myotube ribosome pellets. Data in Figure 7 also show high correlations to 18S + 28S rRNA concentrations in myotubes and mouse muscle tissue. Hence, we contend that these findings support that the RiboAb technique could be used as a surrogate of cell/tissue ribosome content. However, we temper our enthusiasm for various reasons. First, providing data from RiboAb western blotting would be redundant in scenarios whereby RNA and protein samples were being isolated in tandem given that researchers could simply extrapolate cell/tissue ribosome content using UV–Vis. Second, several ribosomal proteins have been shown to exhibit extra‐ribosomal functions and/or co‐localize with other proteins/complexes aside from ribosomes (Warner & McIntosh, 2009); hence, a marginal portion of the RiboAb signal may be non‐specific. Finally, we posit that the RiboAb technique needs to be validated across other models before being widely adopted. Notwithstanding, assuming RiboAb accurately detects muscle ribosome content, we propose potential future utility with this method. For instance, future research could be performed determining if RiboAb immunohistochemistry could be reliably used to ascertain how hypertrophy or atrophy affects the RiboAb signal across multiple fiber types. Moreover, studies employing tissue fractionation techniques (e.g., sarcolemmal isolation, nuclear isolation, and/or sarcoplasmic versus myofibrillar isolation) may be able to utilize the RiboAb method to examine if the subcellular localization of ribosomes is affected under various experimental conditions.

## 5 CONCLUSIONS

These data support that total RNA concentration data yielded from the UV–Vis, fluorometry only, or fluorometry in tandem with microfluidic gel electrophoresis techniques are viable surrogates for cellular/tissue ribosome content. Moreover, preliminary data with the novel RiboAb cocktail indicates that these data may be a surrogate for cell/tissue ribosome content, and future efforts are needed to examine the veracity of using the RiboAb cocktail for immunohistochemistry or tissue fractionation experiments.

## AUTHOR CONTRIBUTIONS

Conceptualization, J.S.G., J.M.M. C.B.M., G.A.N., and M.D.R; funding acquisition, C.M.L. and M.D.R.; investigation and methodology, J.S.G., J.M.M., and M.D.R.; formal analysis, J.S.G. and M.D.R.; supervision, M.D.R., writing‐original draft, J.S.G. and M.D.R.; review and editing, all co‐authors; final approval of manuscript, all co‐authors.

## FUNDING INFORMATION

Funding for assay development and study reagents was provided through the laboratory gift from Nutrabolt (Austin, TX, USA).

## CONFLICT OF INTEREST STATEMENT

M.D.R. has received an unrestricted three‐year laboratory donation from Nutrabolt. M.D.R. M.D.R. has performed industry and commodity‐based contract work, with recent support being received by the US National Dairy Council, The US Peanut Institute, Brickhouse Nutrition, Compound Solutions, The Center for Applied Health Sciences, and Nutrabolt. M.D.R. also performs consulting for personal fees with industry partners in accordance with Auburn University's faculty consulting and annual disclosure policies. None of the other co‐authors have apparent conflicts of interest to report.

## ETHICS STATEMENT

Mouse experiments were conducted in accordance with the institutional guidelines for the care and use of laboratory animals as approved by the Animal Care and Use Committee of the University of Kentucky (protocol #: 2008–0291).

## Data Availability

Raw data related to the current study outcomes will be provided upon reasonable request by emailing the corresponding author (mdr0024@auburn.edu).

## References

[phy270173-bib-0001] Adams, G. R. , Cheng, D. C. , Haddad, F. , & Baldwin, K. M. (2004). Skeletal muscle hypertrophy in response to isometric, lengthening, and shortening training bouts of equivalent duration. Journal of Applied Physiology (1985), 96(5), 1613–1618. 10.1152/japplphysiol.01162.2003 15075307

[phy270173-bib-0002] Chaillou, T. , Kirby, T. J. , & McCarthy, J. J. (2014). Ribosome biogenesis: Emerging evidence for a central role in the regulation of skeletal muscle mass. Journal of Cellular Physiology, 229(11), 1584–1594. 10.1002/jcp.24604 24604615 PMC4868551

[phy270173-bib-0003] Figueiredo, V. C. , Caldow, M. K. , Massie, V. , Markworth, J. F. , Cameron‐Smith, D. , & Blazevich, A. J. (2015). Ribosome biogenesis adaptation in resistance training‐induced human skeletal muscle hypertrophy. American Journal of Physiology. Endocrinology and Metabolism, 309(1), E72–E83. 10.1152/ajpendo.00050.2015 25968575

[phy270173-bib-0004] Figueiredo, V. C. , & McCarthy, J. J. (2019). Regulation of ribosome biogenesis in skeletal muscle hypertrophy. Physiology (Bethesda), 34(1), 30–42. 10.1152/physiol.00034.2018 30540235 PMC6383632

[phy270173-bib-0005] Godwin, J. S. , Michel, J. M. , Ludlow, A. T. , Fruge, A. D. , Mobley, C. B. , Nader, G. A. , & Roberts, M. D. (2024). Relative rDNA copy number is not associated with resistance training‐induced skeletal muscle hypertrophy and does not affect myotube anabolism in vitro. American Journal of Physiology. Regulatory, Integrative and Comparative Physiology, 327(3), R338–R348. 10.1152/ajpregu.00131.2024 39005083 PMC12312789

[phy270173-bib-0006] Green, R. , & Noller, H. F. (1997). Ribosomes and translation. Annual Review of Biochemistry, 66, 679–716. 10.1146/annurev.biochem.66.1.679 9242921

[phy270173-bib-0007] Hammarstrom, D. , Ofsteng, S. J. , Jacobsen, N. B. , Flobergseter, K. B. , Ronnestad, B. R. , & Ellefsen, S. (2022). Ribosome accumulation during early phase resistance training in humans. Acta Physiologica (Oxford, England), 235(1), e13806. 10.1111/apha.13806 35213791 PMC9540306

[phy270173-bib-0008] Haun, C. T. , Vann, C. G. , Mobley, C. B. , Osburn, S. C. , Mumford, P. W. , Roberson, P. A. , Romero, M. A. , Fox, C. D. , Parry, H. A. , Kavazis, A. N. , Moon, J. R. , Young, K. C. , & Roberts, M. D. (2019). Pre‐training skeletal muscle fiber size and predominant fiber type best predict hypertrophic responses to 6 weeks of resistance training in previously trained Young men. Frontiers in Physiology, 10, 297. 10.3389/fphys.2019.00297 30971942 PMC6445136

[phy270173-bib-0009] Hirsch, C. A. (1967). Quantitative determination of the ribosomal ribonucleic acid content of liver and Novikoff hepatoma from fed and from fasted rats. Journal of Biological Chemistry, 242(12), 2822–2827. 4290865

[phy270173-bib-0010] Jiao, L. , Liu, Y. , Yu, X. Y. , Pan, X. , Zhang, Y. , Tu, J. , Song, Y. H. , & Li, Y. (2023). Ribosome biogenesis in disease: New players and therapeutic targets. Signal Transduction and Targeted Therapy, 8(1), 15. 10.1038/s41392-022-01285-4 36617563 PMC9826790

[phy270173-bib-0011] Kim, H. G. , Guo, B. , & Nader, G. A. (2019). Regulation of ribosome biogenesis during skeletal muscle hypertrophy. Exercise and Sport Sciences Reviews, 47(2), 91–97. 10.1249/JES.0000000000000179 30632998

[phy270173-bib-0012] Lee, S. , & Kim, Y. (2022). Ribosome preparation from turquoise killifish skeletal muscle for cryo‐EM. STAR Protocols, 3(1), 101087. 10.1016/j.xpro.2021.101087 35072116 PMC8761774

[phy270173-bib-0013] Makhnovskii, P. A. , Zgoda, V. G. , Bokov, R. O. , Shagimardanova, E. I. , Gazizova, G. R. , Gusev, O. A. , Lysenko, E. A. , Kolpakov, F. A. , Vinogradova, O. L. , & Popov, D. V. (2020). Regulation of proteins in human skeletal muscle: The role of transcription. Scientific Reports, 10(1), 3514. 10.1038/s41598-020-60578-2 32103137 PMC7044165

[phy270173-bib-0014] McCarthy, J. J. , Mula, J. , Miyazaki, M. , Erfani, R. , Garrison, K. , Farooqui, A. B. , Srikuea, R. , Lawson, B. A. , Grimes, B. , Keller, C. , Van Zant, G. , Campbell, K. S. , Esser, K. A. , Dupont‐Versteegden, E. E. , & Peterson, C. A. (2011). Effective fiber hypertrophy in satellite cell‐depleted skeletal muscle. Development, 138(17), 3657–3666. 10.1242/dev.068858 21828094 PMC3152923

[phy270173-bib-0015] Mesquita, P. H. C. , Vann, C. G. , Phillips, S. M. , McKendry, J. , Young, K. C. , Kavazis, A. N. , & Roberts, M. D. (2021). Skeletal muscle ribosome and mitochondrial biogenesis in response to different exercise training modalities. Frontiers in Physiology, 12, 725866. 10.3389/fphys.2021.725866 34646153 PMC8504538

[phy270173-bib-0016] Michel, J. M. , Godwin, J. S. , Plotkin, D. L. , Mesquita, P. H. C. , McIntosh, M. C. , Ruple, B. A. , Libardi, C. A. , Mobley, C. B. , Kavazis, A. N. , & Roberts, M. D. (2023). Proteolytic markers associated with a gain and loss of leg muscle mass with resistance training followed by high‐intensity interval training. Experimental Physiology, 108(10), 1268–1281. 10.1113/EP091286 37589512 PMC10543615

[phy270173-bib-0017] Moberg, M. , Hendo, G. , Jakobsson, M. , Mattsson, C. M. , Ekblom‐Bak, E. , Flockhart, M. , Ponten, M. , Soderlund, K. , & Ekblom, B. (2017). Increased autophagy signaling but not proteasome activity in human skeletal muscle after prolonged low‐intensity exercise with negative energy balance. Physiological Reports, 5(23), e13518. 10.14814/phy2.13518 29208687 PMC5727276

[phy270173-bib-0018] Mobley, C. B. , Haun, C. T. , Roberson, P. A. , Mumford, P. W. , Kephart, W. C. , Romero, M. A. , Osburn, S. C. , Vann, C. G. , Young, K. C. , Beck, D. T. , Martin, J. S. , Lockwood, C. M. , & Roberts, M. D. (2018). Biomarkers associated with low, moderate, and high vastus lateralis muscle hypertrophy following 12 weeks of resistance training. PLoS One, 13(4), e0195203. 10.1371/journal.pone.0195203 29621305 PMC5886420

[phy270173-bib-0019] Nader, G. A. , McLoughlin, T. J. , & Esser, K. A. (2005). mTOR function in skeletal muscle hypertrophy: Increased ribosomal RNA via cell cycle regulators. American Journal of Physiology. Cell Physiology, 289(6), C1457–C1465. 10.1152/ajpcell.00165.2005 16079186

[phy270173-bib-0020] Nakada, S. , Ogasawara, R. , Kawada, S. , Maekawa, T. , & Ishii, N. (2016). Correlation between ribosome biogenesis and the magnitude of hypertrophy in overloaded skeletal muscle. PLoS One, 11(1), e0147284. 10.1371/journal.pone.0147284 26824605 PMC4732984

[phy270173-bib-0021] O'Reilly, J. , Ono‐Moore, K. D. , Chintapalli, S. V. , Rutkowsky, J. M. , Tolentino, T. , Lloyd, K. C. K. , Olfert, I. M. , & Adams, S. H. (2021). Sex differences in skeletal muscle revealed through fiber type, capillarity, and transcriptomics profiling in mice. Physiological Reports, 9(18), e15031. 10.14814/phy2.15031 34545692 PMC8453262

[phy270173-bib-0022] Roberts, M. D. , McCarthy, J. J. , Hornberger, T. A. , Phillips, S. M. , Mackey, A. L. , Nader, G. A. , Boppart, M. D. , Kavazis, A. N. , Reidy, P. T. , Ogasawara, R. , Libardi, C. A. , Ugrinowitsch, C. , Booth, F. W. , & Esser, K. A. (2023). Mechanisms of mechanical overload‐induced skeletal muscle hypertrophy: Current understanding and future directions. Physiological Reviews, 103(4), 2679–2757. 10.1152/physrev.00039.2022 37382939 PMC10625844

[phy270173-bib-0023] Roberts, M. D. , Ruple, B. A. , Godwin, J. S. , McIntosh, M. C. , Chen, S. Y. , Kontos, N. J. , Agyin‐Birikorang, A. , Michel, M. , Plotkin, D. L. , Mattingly, M. L. , Mobley, B. , Ziegenfuss, T. N. , Fruge, A. D. , & Kavazis, A. N. (2024). A novel deep proteomic approach in human skeletal muscle unveils distinct molecular signatures affected by aging and resistance training. Aging (Albany NY), 16(8), 6631–6651. 10.18632/aging.205751 38643460 PMC11087122

[phy270173-bib-0024] Stec, M. J. , Kelly, N. A. , Many, G. M. , Windham, S. T. , Tuggle, S. C. , & Bamman, M. M. (2016). Ribosome biogenesis may augment resistance training‐induced myofiber hypertrophy and is required for myotube growth in vitro. American Journal of Physiology. Endocrinology and Metabolism, 310(8), E652–E661. 10.1152/ajpendo.00486.2015 26860985 PMC4835943

[phy270173-bib-0025] Tanaka, K. (2009). The proteasome: Overview of structure and functions. Proceedings of the Japan Academy. Series B, Physical and Biological Sciences, 85(1), 12–36. 10.2183/pjab.85.12 19145068 PMC3524306

[phy270173-bib-0026] von Walden, F. , Casagrande, V. , Ostlund Farrants, A. K. , & Nader, G. A. (2012). Mechanical loading induces the expression of a pol I regulon at the onset of skeletal muscle hypertrophy. American Journal of Physiology. Cell Physiology, 302(10), C1523–C1530. 10.1152/ajpcell.00460.2011 22403788

[phy270173-bib-0027] von Walden, F. , Liu, C. , Aurigemma, N. , & Nader, G. A. (2016). mTOR signaling regulates myotube hypertrophy by modulating protein synthesis, rDNA transcription, and chromatin remodeling. American Journal of Physiology. Cell Physiology, 311(4), C663–C672. 10.1152/ajpcell.00144.2016 27581648

[phy270173-bib-0028] Warner, J. R. , & McIntosh, K. B. (2009). How common are extraribosomal functions of ribosomal proteins? Molecular Cell, 34(1), 3–11. 10.1016/j.molcel.2009.03.006 19362532 PMC2679180

[phy270173-bib-0029] Weir, J. P. (2005). Quantifying test‐retest reliability using the intraclass correlation coefficient and the SEM. Journal of Strength and Conditioning Research, 19(1), 231–240. 10.1519/15184.1 15705040

[phy270173-bib-0030] Wen, Y. , Vechetti, I. J., Jr. , Valentino, T. R. , & McCarthy, J. J. (2020). High‐yield skeletal muscle protein recovery from TRIzol after RNA and DNA extraction. BioTechniques, 69(4), 264–269. 10.2144/btn-2020-0083 32777951 PMC7566772

